# Using DNA metabarcoding for simultaneous inference of common vampire bat diet and population structure

**DOI:** 10.1111/1755-0998.12891

**Published:** 2018-05-16

**Authors:** Kristine Bohmann, Shyam Gopalakrishnan, Martin Nielsen, Luisa dos Santos Bay Nielsen, Gareth Jones, Daniel G. Streicker, M. Thomas P. Gilbert

**Affiliations:** ^1^ Section for Evolutionary Genomics Natural History Museum of Denmark University of Copenhagen Copenhagen Denmark; ^2^ School of Biological Sciences University of East Anglia Norwich Norfolk UK; ^3^ School of Biological Sciences University of Bristol Bristol UK; ^4^ Institute of Biodiversity, Animal Health and Comparative Medicine MRC‐University of Glasgow Centre for Virus Research University of Glasgow Glasgow UK; ^5^ NTNU University Museum Trondheim Norway

**Keywords:** diet analyses, ecological genetics, environmental DNA, mammals, predator–prey interactions, wildlife management

## Abstract

Metabarcoding diet analysis has become a valuable tool in animal ecology; however, co‐amplified predator sequences are not generally used for anything other than to validate predator identity. Exemplified by the common vampire bat, we demonstrate the use of metabarcoding to infer predator population structure alongside diet assessments. Growing populations of common vampire bats impact human, livestock and wildlife health in Latin America through transmission of pathogens, such as lethal rabies viruses. Techniques to determine large‐scale variation in vampire bat diet and bat population structure would empower locality‐ and species‐specific projections of disease transmission risks. However, previously used methods are not cost‐effective and efficient for large‐scale applications. Using bloodmeal and faecal samples from common vampire bats from coastal, Andean and Amazonian regions of Peru, we showcase metabarcoding as a scalable tool to assess vampire bat population structure and feeding preferences. Dietary metabarcoding was highly effective, detecting vertebrate prey in 93.2% of the samples. Bats predominantly preyed on domestic animals, but fed on tapirs at one Amazonian site. In addition, we identified arthropods in 9.3% of samples, likely reflecting consumption of ectoparasites. Using the same data, we document mitochondrial geographic population structure in the common vampire bat in Peru. Such simultaneous inference of vampire bat diet and population structure can enable new insights into the interplay between vampire bat ecology and disease transmission risks. Importantly, the methodology can be incorporated into metabarcoding diet studies of other animals to couple information on diet and population structure.

## 1 INTRODUCTION

Vampire bats occur in Central and South America and are exceptional among mammals in that they subsist exclusively on blood (Greenhall, Joermann, & Schmidt, 1983; Greenhall, Schmidt, & Joermann, 1984; Greenhall & Schutt, 1996). The common vampire bat (*Desmodus rotundus*) is one of three extant species of vampire bats (Chiroptera, Phyllostomidae, Desmodontinae) (Greenhall et al., 1983). Before the introduction of domestic animals, common vampire bat populations in Latin America were likely small, as the bats’ only source of bloodmeals presumably was large wild animals. However, the proliferation of domestic animals following European colonization created an abundant and reliable food source, which seems to support larger population sizes of vampire bats (Costa & Esbérard, 2011; Delpietro, Marchevsky, & Simonetti, 1992; Fenton et al., 1992; Voigt & Kelm, 2006).

Large populations of common vampire bats impact both human and animal health. Vampire bats are the primary reservoir of human and livestock rabies in Latin America, a virus which causes a universally lethal encephalitis and has a host range spanning all mammals (Delpietro, Lord, Russo, & Gury‐Dhomen, 2009; Salmón‐Mulanovich et al., 2009; Schneider et al., 2009; Warner et al., 1999). Vampire bats may also transmit trypanosome parasites to domestic horses and cattle (reviewed by Hoare (1965)) and act as reservoirs and potential vectors of pathogenic bacteria and viruses (Bai et al., 2012; Brandão et al., 2008; Lima et al., 2013; Sabino‐Santos et al., 2015; Volokhov et al., 2017). Although culling of vampire bats is a widespread practice for rabies control, recent work has questioned its efficacy and cost‐effectiveness and instead advocated for vaccination of susceptible species and ecological strategies to prevent exposures (Anderson et al., 2014; Blackwood, Streicker, Altizer, & Rohani, 2013). Appropriate use of either strategy relies critically on knowledge of local vampire bat–prey interactions; however, existing methodologies to study vampire bat diet are limited. Stable isotope analyses of bat tissues have been commonly applied and can distinguish between domestic livestock and wildlife, but are unable to distinguish prey at the species level (Streicker & Allgeier, 2016; Voigt & Kelm, 2006). Other studies using DNA markers have been more specific, but failed to identify prey in large proportions of samples and used time‐consuming approaches that do not easily scale to large data sets (Bobrowiec, Lemes, & Gribel, 2015; Carter, Coen, Stenzler, & Lovette, 2006; Ito, Bernard, & Torres, 2016). This illustrates that while genetic approaches offer an attractive solution to reconstruct vampire bat diets with high specificity, existing methods have not yet reached a technical level that would make them cost‐effective and efficient for large‐scale applications.

A second challenge for managing vampire bat‐transmitted rabies is that transmission to humans, wildlife and livestock is underpinned by metapopulation dynamics and travelling waves of infection within vampire bats (Benavides, Valderrama, & Streicker, 2016; Blackwood et al., 2013). These complex spatial dynamics challenge prediction of localities at risk of rabies outbreaks; however, recent work has shown that genetic inference of vampire bat population structure can identify the future geographic pathways of ongoing epizootics (Streicker et al., 2016). Genetic tools for simultaneous inference of vampire bat diet and population structure are therefore desirable to forecast which areas rabies is likely to invade next, and the species that will be at risk of spillover infection. Such information could together guide the timing and species to be targeted for vaccination. Moreover, tools for large‐scale analysis of bat diet would help to forecast changes in human rabies risk following deforestation or hunting‐driven declines in preferred prey or conversely, how recovery of wildlife due to conservation efforts could reduce human rabies.

One potential solution to the technical limitations of previous studies of vampire bat diet is to draw on recently developed advances in biodiversity assessment linked to environmental DNA (eDNA), where second‐generation sequencing is applied to DNA extracted from environmental samples such as soil, water, gut contents and faeces (Bohmann et al., 2014; Taberlet, Coissac, Hajibabaei, & Rieseberg, 2012a). Ideally, this approach would be applied to samples collected from vampire bat roost environments, enabling analyses to be conducted over larger geographic scales without the need for time‐consuming capture and sampling of wild bats. Today, the principal approach for sequencing informative DNA in eDNA mixtures is the so‐called DNA metabarcoding approach, in which 5′‐nucleotide‐tagged primers (Binladen et al., 2007) are used to PCR amplify mitochondrial mini‐barcodes of taxa within a taxonomic group (Taberlet, Coissac, & Pompanon, 2012b). The amplicons are subsequently sequenced in parallel on a second‐generation sequencing platform. After processing the resulting sequences, taxa can be identified by comparing the obtained “barcode” sequences to DNA reference databases (Valentini et al., 2009). The ability to only sequence informative DNA markers and to process and sequence many samples in parallel make metabarcoding a cost‐effective and efficient way to assess biodiversity. Furthermore, if the DNA reference database and chosen barcode marker allow it, assignments can be made for prey down to the species level. Given this, metabarcoding has experienced increasing popularity and is now used in biodiversity assessment (e.g., Chariton, Court, Hartley, Colloff, & Hardy, 2010) and animal diet studies (e.g., Bohmann et al., 2011; Deagle, Kirkwood, & Jarman, 2009; Soininen et al., 2009). Metabarcoding has recently been applied to determine hosts of pathogen‐transmitting invertebrate blood‐feeders, for example, mosquitoes (Logue et al., 2016) and sand flies (Kocher et al., 2017). Metabarcoding of vampire bat diet, however, represents a distinct methodological challenge, compared to, for example carnivores or generalist predators, as the primers that target mammalian prey will inevitably also amplify bat DNA. In such studies, predator sequences are in excess to a point where they can prevent detection of prey taxa (e.g., Deagle, Eveson, & Jarman, 2006; Shehzad et al., 2012). To optimize detection of prey taxa, they are often reduced through presequencing measures, such as predator blocking primers (Deagle et al., 2009; Vestheim & Jarman, 2008). Post‐sequencing, remaining predator sequences are used to validate predator species identity after which they are discarded (e.g., Piñol, San Andrés, Clare, Mir, & Symondson, 2014). Co‐amplified predator sequences might, however, offer an overlooked opportunity to get insights into predator population structure simultaneously with the diet analysis.

In this study, we aim to determine whether metabarcoding diet analyses can be used to simultaneously study predator population structure and to evaluate metabarcoding as a method for large‐scale common vampire bat–prey detection at the species level. In doing so, we assess common vampire bat prey and population structure across the common vampire bat's range in Peru.

## 2 MATERIALS AND METHODS

**Figure 1:**
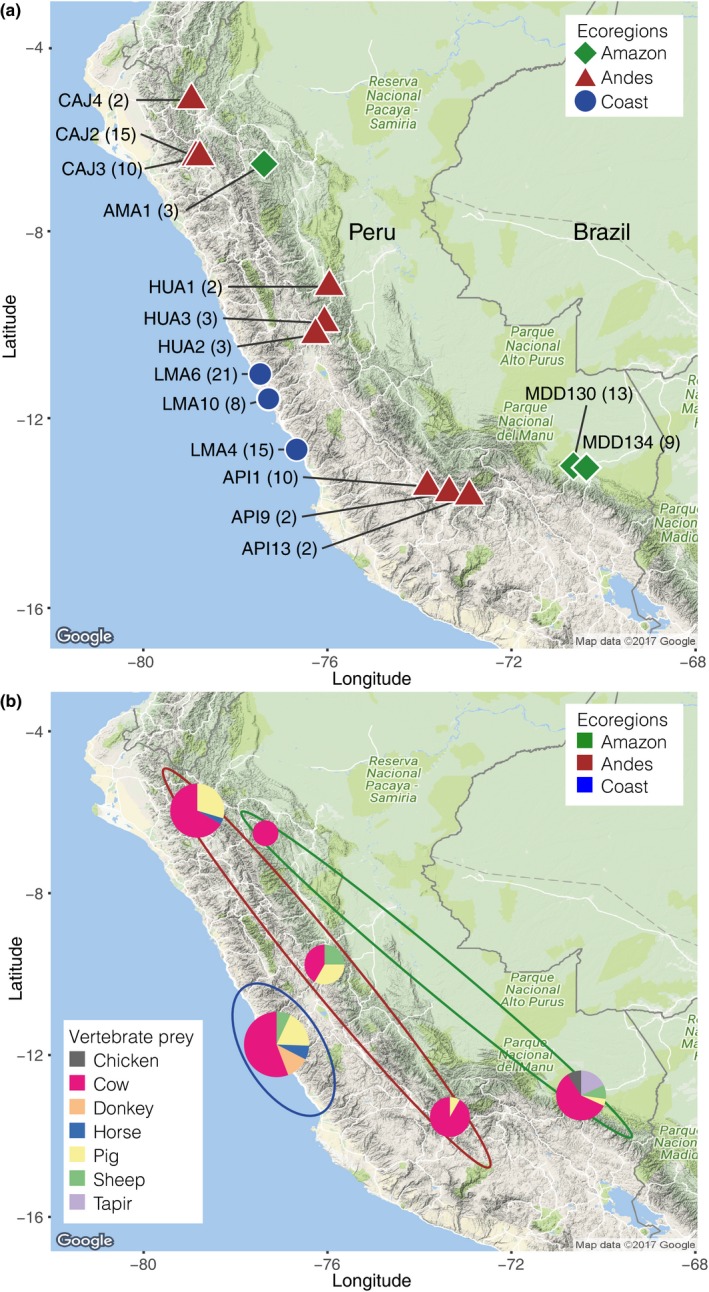
Collection sites and identified diet. (a) Overview of 15 collection sites for common vampire bat bloodmeal and faecal samples spanning three ecoregions in Peru. Sample numbers are listed in parentheses. (b) Vertebrate prey identified through metabarcoding analyses in six areas spanning the 15 collection sites. Pie charts are scaled to reflect sample size. Ecoregions are shown by ellipses

Bloodmeal and faecal samples were collected from common vampire bats (*Desmodus rotundus*) caught between 2009 and 2013 at 15 sites across three ecoregions in Peru; Andes, Amazon and Pacific coast (Figure 1a; Table S1a). Bat capture and bloodmeal sampling followed Streicker and Allgeier (2016). Bats were captured using mist nets and/or harp traps placed outside daytime roosts (caves, mines, tunnels and hollow trees) between 18:00 and 06:00 hr. Bats were caught at daytime roosts to avoid biasing dietary inferences by sampling bats near potential prey (Bobrowiec et al., 2015). Captured bats were anaesthetized by intramuscular injection of ketamine (83–125 mg/kg), and a sterile 5‐French nasogastric feeding tube attached to an empty syringe was inserted through the oesophagus into the stomach to extract a sample from the bloodmeal. For each bat, ca. 50 μl blood was extracted and expelled onto a Whatman Flinders Technology Associates (FTA) card and desiccated. Bats were kept in cloth bags until they recovered from the anaesthesia after which they were released. There were no mortality or signs of injury following bloodmeal collection. Faecal samples from individual bats were collected from cloth bags or during handling, while pooled faecal samples (~5) were collected opportunistically from underneath roosting bats and stored in RNAlater (Sigma).

In total, 118 common vampire bat samples were analysed; 110 bloodmeals, five individual faecal samples and three pooled faecal samples (Table S1a). In addition, three bloodmeal samples from hairy‐legged vampire bat (*Diphylla ecaudata*) collected in the MDD134 site were processed. Details for these can be found in Supporting Information S2. DNA extractions were carried out in a dedicated pre‐PCR laboratory, and PCR mixes were set up in a dedicated no‐DNA laboratory to minimize risk of contamination. Filter tips were used for pipetting, and negative controls were included in all steps.

For bloodmeal samples, three punches of 3‐mm diameter were collected from FTA cards and extracted using the Qiagen Investigator Kit (protocol: Isolation of DNA from fta and guthrie cards, version 2). Faecal samples were extracted with the Qiagen Mini Stool Kit (protocol: Isolation of DNA from stool for pathogen detection (06/2012 version)).

DNA metabarcoding was carried out using a set of mammalian mitochondrial 16s rRNA‐targeting primers (16smam1/16smam2) (Taylor, 1996) amplifying a ca. 95‐bp fragment (excluding primers) and a set of metazoan mitochondrial COI‐targeting primers (mlCOIintF/jgHCO2198) amplifying a partial fragment (313 bp excl. primers) of the commonly used COI barcode region (Geller, Meyer, Parker, & Hawk, 2013; Leray et al., 2013). The primers are from here on referred to as 16s and COI. Both primer sets were 5′ nucleotide tagged with 6–8 nucleotide tags (Binladen et al., 2007).

Prior to metabarcoding PCRs, a subset of DNA extracts were prescreened using SYBR Green qPCR (Murray, Coghlan, & Bunce, 2015; Schnell, Bohmann, & Gilbert, 2015; Shapiro & Hofreiter, 2012) with both primer sets on a dilution series of a subset of the sample extracts and on undiluted extraction negative controls. This enabled (i) screening for contamination in negative extraction controls and across samples, (ii) determination of the optimal cycle number in the subsequent tagged PCR amplifications that would stop the reaction during the exponential phase or just after the plateau and (iii) determination of the maximal amount of template in which PCR inhibitory substances would not distort the results of amplification of prey DNA. The qPCRs were carried out in 25 μl reactions containing 1 μl template DNA, 1 U AmpliTaq Gold, 1× Gold PCR Buffer and 2.5 mm MgCl_2_ (all from Applied Biosystems), 0.2 mm dNTP Mix (Invitrogen), 0.4 μm each of 5′ nucleotide tagged forward and reverse primer and 1 μl of SYBR Green/ROX solution (one part SYBR Green I nucleic acid gel stain (S7563) (Invitrogen), four parts ROX Reference Dye (12223‐012) (Invitrogen) and 2000 parts high grade DMSO). The 16s amplifications were carried out with the following parameters: 95°C for 5 min, followed by 40 cycles of 95°C for 12 s, 59°C for 30 s and 70°C for 25 s and followed by a dissociation curve. The COI amplifications were carried out with the following parameters: 95°C for 5 min, followed by 40 cycles of 95°C for 15 s, 51°C for 30 s and 72°C for 60 s and followed by a dissociation curve. For both primer sets, amplification plots indicated that using 1 μl of neat extract and running 25 cycles were optimal across samples for the subsequent PCR amplification. Amplification and dissociation curves confirmed that only primer–dimers, and not prey DNA, were present in the negative extraction controls.

For each primer set, tagged PCRs were carried out with three PCR replicates for each sample extract, extraction negative control and each of three positive controls. PCR amplifications were performed with matching tags (e.g., F1‐R1, F2‐R2, etc.) to ensure that tag jumps would not result in false assignments of sequences to samples (Schnell et al., 2015). Furthermore, the three PCR replicates from each sample were made with different tag combinations to account for possible tagged primer biases and cross‐contamination (Berry, Ben Mahfoudh, Wagner, & Loy, 2011; Schnell et al., 2015). PCRs were prepared as the qPCR, although omitting SYBR Green/ROX, adding a final extension of 7 min and omitting the dissociation segment. All negative controls appeared negative when visualizing PCR products on 2% agarose gels. DNA extracts that initially failed to PCR amplify were re‐attempted with 30 cycles. Only PCR products with different tags were pooled to enable sequencing of many PCR replicates in parallel, while still being able to track the tagged PCR products back to the correct PCR replicate. Amplicon pools were bead‐purified (Faircloth & Glenn, 2014; Rohland & Reich, 2012) and quantified on a 2100 Bioanalyzer (Agilent Technologies). Library preparations were carried out principally following Schnell et al. (2015), although omitting size selection. Optimal cycle number for the index‐PCR was estimated using qPCR, and the libraries were indexed with eight cycle PCRs and bead‐purified. Each indexed library was quantified on a 2100 Bioanalyzer, after which they were pooled and sequenced with 230‐bp paired‐end chemistry on an Illumina MiSeq sequencing platform aiming at ca. 15,000 paired reads per PCR replicate.

Sequence reads were initially trimmed for adapters, consecutive stretches of N's and low‐quality bases, after which paired‐end reads were merged using adapterremoval version 2 (Schubert, Lindgreen, & Orlando, 2016). Sequences within each library were sorted according to primer and tag combinations using a modified version of DAMe (https://github.com/shyamsg/DAMe; Zepeda‐Mendoza, Bohmann, Carmona Baez, & Gilbert, 2016). For each primer set, thresholds for filtering sequences across each sample's PCR replicates followed a restrictive approach and were guided by the negative and positive controls sequenced (Alberdi, Aizpurua, Gilbert, & Bohmann, 2018; De Barba et al., 2014). Sequences were clustered using sumaclust (Mercier, Boyer, Bonin, & Coissac, 2013) with a similarity score of 0.96 and a maximum abundance ratio of 0.90. A contingency table of operational taxonomic units (OTUs) by samples was created for each primer set, and copy numbers of OTUs were normalized across samples. blastn (Altschul et al., 1997) was used to compare the OTUs against the ncbi genbank database (http://www.ncbi.nlm.nih.gov/), and the output was imported into megan community edition version 6.5 (Huson, Auch, Qi, & Schuster, 2007). OTUs assigned to ascomycetes were removed from the COI data set. In the contingency table, OTUs were grouped into categories according to assigned taxonomy: common vampire bat, vertebrate prey and for COI, invertebrates. Within each of the categories, the proportion of sequences of each OTU was calculated in each sample. Within OTUs assigned to common vampire bats, vampire bat assignments to samples were made if an OTU had >95% (16s) and >99% (COI) of sequences assigned to vampire bat OTUs in the sample (erroneous OTUs were in lower frequency for the COI marker). For vertebrate prey identifications, a conservative approach for removing erroneous OTUs was used. Initially, OTUs that never had >5% of sequences in any sample within the vertebrate category were removed. Following this, vertebrate prey OTUs were assigned to samples if they had >10% of sequences (bloodmeals and individual faecal samples) and >0.5% of sequences (pooled faecal samples) of sequences within the vertebrate prey category. Only one invertebrate OTU was identified, and all assignments of this OTU were retained.

Taxonomic assignments of OTUs assigned to samples were made with either primer set, and the ncbi genbank database (http://www.ncbi.nlm.nih.gov/) and barcode of life database (bold; Ratnasingham & Hebert, 2007), were used for taxonomic assignment of the 16s and COI OTUs, respectively. Species assignments were made if both markers had 100% match to only the same species, and genus assignment if either marker had 100% matches to several species within the same genus. For invertebrates, remaining OTUs generally had poor reference database coverage, and identifications were made to taxonomic family level or higher taxonomic levels against all barcode records in bold. For each overall area, vampire bat vertebrate prey was visualized as pie charts on a map made in ggmap version 2.7.

To assess intraspecific variation in the common vampire bats, sequences from both primer sets were clustered with sumaclust (Mercier et al., 2013) at 98.7% similarity, which was found to balance clustering of erroneous sequences (e.g., arisen due to PCR and sequencing errors) with retrieval of intraspecific diversity. The filtered amplicon sequences for each sample were mapped back using bwa (version 0.7.15) (Li & Durbin, 2009) to the OTU centres detected using sumaclust. The filtered reads that mapped to the OTUs assigned to common vampire bat were used to explore the intraspecific diversity for each marker. Within samples, OTU assignments with >2% of all sequences assigned to the sample were retained, and presence–absence of the 16s and COI common vampire bat OTUs were spatially visualized using ggmap version 2.

Prey availability was calculated based on the livestock density, in a 0.00833° latitude × 0.00833° longitude rectangle, obtained from GEO wiki (http://www.livestock.geo-wiki.org). For each site, the prey availability was computed by averaging the densities in a 5 × 5 square grid around the sampling site. The choice of the size of the grid around each site was guided by the observation in a previous study that the common vampire bats fly a nightly distance of 3.4 km on average (Wilkinson, 1985). At this latitude, the 5 × 5 grid corresponds approximately to a 4.5 km × 4.5 km square. For each area, the prey densities were calculated by taking a union of the grid sites for each sampling site in the area.

Prey preference was tested using the prey densities computed for each area. As the prey densities were available for only a subset of the prey species detected in our data, viz. cow, chicken, pig and sheep, the prey preference analysis was limited to these species. For each area and for all areas combined, the expected number of each of these prey was computed using the prey densities in that area and the total number of prey detections. The number of observed prey in each area was calculated as the number of prey detections using metabarcoding. Subsequently, prey preference was calculated using a Pearson's chi‐square goodness of fit test, testing for agreement between the observed and the expected numbers of prey individuals from each prey species. Note that the results should be interpreted with care, given the low expected numbers of some prey species. As the number of chicken prey detections was low (two total detections combined across all sites), the prey preference was computed for only mammalian prey species (cow, pig, sheep), using the same method as described above.

## 3 RESULTS

### 3.1 Primer performance

**Figure 2:**
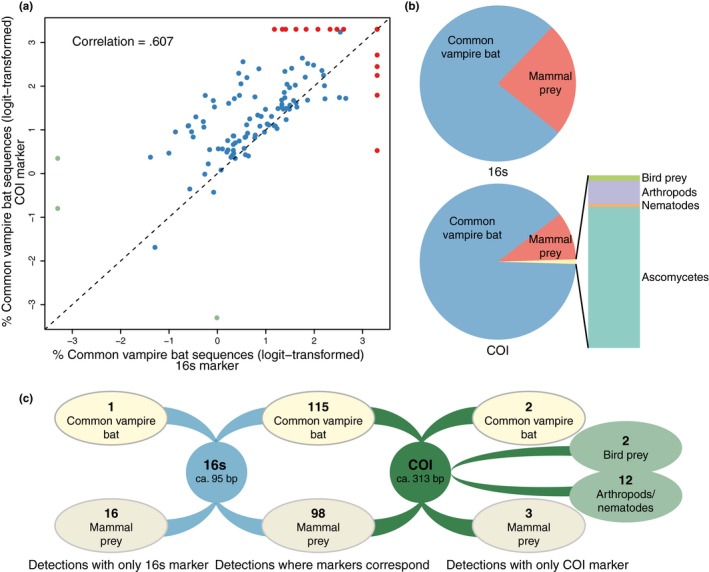
Performance of two primer sets, mammal 16s and metazoan COI, for common vampire bat diet assessment. (a) Scatter plot of the logit‐transformed percentages of common vampire bat sequences detected with the two primer sets. Each data point represents one common vampire bat bloodmeal or faecal sample (*n* = 118). The *x* = *y* line is shown in dashed black. Data points for samples for which one of the markers had 0% common vampire bat sequences are green. Data points for samples in which one of the markers had 100% common vampire bat sequences are red. The correlation coefficient (*r* ^2^) is computed based on nontransformed data. (b) Proportion of sequences assigned to OTUs in different taxonomic categories in the total data set (118 samples). (c) Number of detections of common vampire bat, mammal prey, bird prey and arthropods/nematodes with the two primer sets in 118 faecal and bloodmeal samples

Primer choice is crucial in metabarcoding studies as the primers principally determine which taxa will get sequenced. Although both the 16s and COI markers detected common vampire bat in almost all samples (98.3% and 99.2%, respectively, Table S1b), the relative abundance of common vampire bat sequences was higher for the longer COI marker both within samples (Figure 2a), and for all samples combined (Figure 2b). As such, fewer prey sequences could be assigned when using the COI primer set (Figure 2b), with obvious consequences for prey detection power. For example, while the 16s primer set detected prey in 90.7% of samples, the COI marker only detected prey in 82.2% of samples (Table S1b). Furthermore, while for the 98 cases in which mammal prey was detected, both markers identified the same taxa, the 16s primers enabled 16 additional mammalian prey detections, while the COI primer set only enabled three additional mammalian prey detections (Figure 2c). This difference was to some extent outweighed by the ability of the COI primer set to detect birds, arthropods and nematodes (Figure 2b,c).

### 3.2 Vertebrate prey

The combined detections of the 16s and COI primer sets identified common vampire bat in all 118 samples, and vertebrate prey in 93.2% of the samples (Table 1). In samples with no vertebrate prey detections, only common vampire bat DNA was detected by the 16s primer set, while the COI primer set either detected only common vampire bat or common vampire bat and ascomycetes. In bloodmeal and individual faecal samples with vertebrate prey detections, generally only a single prey taxon was identified, whereas two or three prey taxa were identified in pooled faecal samples collected from roost environments (Table 1).

**Table 1:** Number of common vampire bat samples in which vampire bat and vertebrate prey were detected through metabarcoding. Furthermore, the number of identified prey taxa in samples with prey detections

	Blood meal samples	Individual faecal samples	Pooled faecal samples	All samples
Analysed samples	110	5	3	118
Vampire bat detection	110 (100%)	5 (100%)	3 (100%)	118 (100%)
Vertebrate prey detection	103 (93.6%)	4 (80%)	3 (100%)	110 (93.2%)
Min.–max. (average) number vertebrate prey taxa in samples with vertebrate prey detections	1–2 (1.04)	1 (1)	2–3 (2.67)	N/A

**Figure 3:**
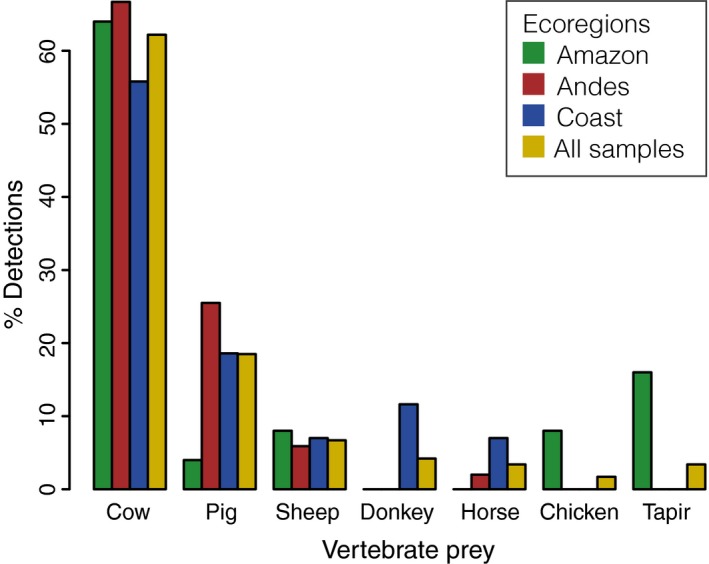
Percentages of common vampire bat vertebrate prey taxa identified within each Peruvian ecoregion and for all samples combined

Cows were the most frequently detected prey species in all sampled areas, regardless of ecoregion (Figures 1b and 3). Pigs and sheep were also preyed upon in all ecoregions, while donkey, horse, chicken and the only wild species detected, tapir, were preyed upon only in specific ecoregions (Figures 1b and 3). Tapir was detected in four of the 13 individual bats from the Amazonian MDD130 site.

A species accumulation curve showed that vertebrate prey detections reached a plateau after around 80 samples after which adding more samples to the analysis did not increase the number of prey species detected (Figure S1a). This suggests that at the time of sampling, the vampire bats in these localities did not rely on a more diverse prey base than what was detected.

For all sites combined, there were fewer chicken detections in the prey than what would be expected based on numbers of chicken available, while cows and pigs were overrepresented in the diet compared with availability (Table S1d). When narrowing prey preference investigations to mammals alone (cow, pig and sheep), more cows were detected than would be expected based on abundance, while sheep were underrepresented (Table S1e).

### 3.3 Invertebrate detections

Operational taxonomic units from five arthropod and one (parasitic) nematode taxon were detected by the COI metazoan primers in twelve common vampire bat samples (Table 2). Arthropod DNA was detected in both bloodmeal and faecal samples in a third of the collection sites, in all three ecoregions and in samples with different vertebrate prey taxa (Table 2). We also detected a nematode in the bloodmeal from a hairy‐legged vampire bat (*Diphylla ecaudata*) (Table S2), suggesting that the ability to detect nematodes likely infecting the bat might not be unique to common vampire bats. As the DNA reference database coverage for the South American arthropods and nematodes is not comprehensive, the taxonomic level of OTU assignments spanned from genus to phylum. However, identifications at the lowest taxonomic levels were to arthropod taxa that are ectoparasites on vertebrates or nematodes (Table 2).

**Table 2:** Arthropod and nematode taxa identified through metabarcoding of common vampire bat faecal extracts using metazoan COI primers

Invertebrate	Taxa	English name	Info	Sample type	Ecoregion	Site	Vertebrate prey detected in sample
Arthropod	Arachnida, Ixodida, Ixodidae, *Rhipicephalus* sp.	Tick	Adults of most species parasitize wild and domestic artiodactyls, perissodactyls or carnivores	Bloodmeal	Amazon	MDD134	Cow
Arthropod	Insecta, Diptera, Hippoboscoidea, Streblidae /Hippoboscidae	Louse flies or bat flies	Ectoparasites of mammals and birds	Pooled faecal	Andes	HUA2	Pig, cow, sheep
Arthropod	Insecta, Diptera			Pooled faecal	Andes	HUA1	Pig, cow, sheep
Arthropod	Insecta			Bloodmeal	Amazon	MDD130	Cow
Arthropod	Undetermined			Bloodmeal	Amazon	MDD130	Tapir
				Bloodmeal	Andes	API1	Cow
				Bloodmeal	Andes	API13	Cow
				Bloodmeal	Coast	LMA10	Cow
				Bloodmeal	Coast	LMA4	Cow
				Bloodmeal	Coast	LMA6	Cow
				Bloodmeal	Coast	LMA6	Donkey
Nematode	Undetermined			Bloodmeal	Amazon	AMA1	Cow

### 3.4 Population structure

**Figure 4:**
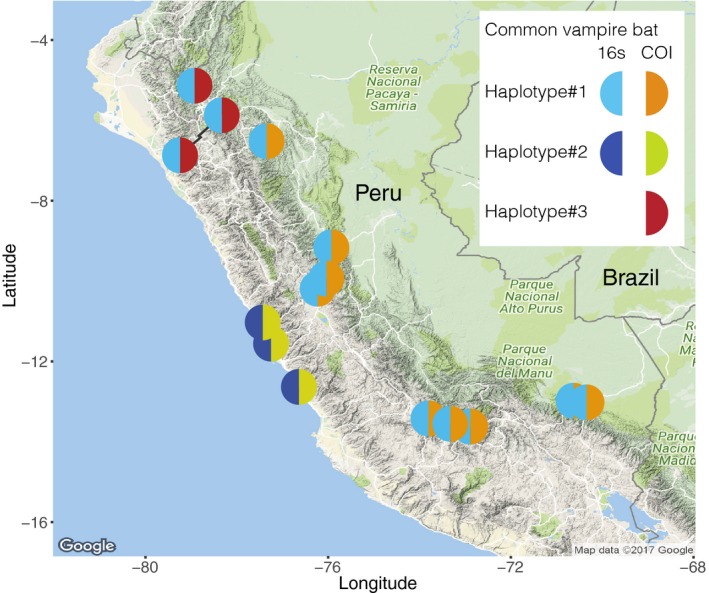
Geographic distribution of common vampire bat 16s and COI haplotypes. Map made in ggmap version 2.7

Intraspecific diversity was identified among common vampire bat mitochondrial sequences for both the 16s and COI marker. Two haplotypes were identified for the 16s marker, and three for the COI marker (Figure 4). The level of observed differentiation was high with 6.5% difference between the two 16s haplotypes (6‐bp difference over the 92‐bp fragment), and 1.92%–7.99% pairwise differences between the three COI haplotypes (6‐ to 25‐bp difference over the 313‐bp fragment) (Figure S1b). Within sampling locations, individual bats had the same haplotypes (Figure 4). Our results show clear phylogeographic structure of the mitochondrial COI and 16s markers in the common vampire bat in Peru: the 16s and COI haplotype 2 were restricted to the coastal sites, while at the nine southern collection sites in the Andes and Amazon, the 16s and COI haplotype 1 co‐occurred in all bats (Figure 4). Bats at the three collection sites in the western slopes of the Andes in northern Peru were the only ones to harbour the third COI haplotype (Figure 4).

## 4 DISCUSSION

Just as in other metabarcoding diet studies where the predator is in the same taxonomic group as the prey, the metabarcoding primers used in this study inevitably also amplified the common vampire bat DNA. However, this co‐amplification allowed us to examine population structure in the common vampire bat alongside identification of diet items. To our knowledge, this is the first time that metabarcoding has been used to simultaneously assess population structure and diet. This has, however, been shown with metagenomics of primate faecal samples (Srivathsan, Ang, Vogler, & Meier, 2016; Srivathsan, Sha, Vogler, & Meier, 2015). As the metagenomics approach relied on shotgun sequencing of total DNA, apart from information on both expected and unexpected diet taxa it yielded whole primate mitogenomes and data on intestinal parasites and bacteria. In contrast, as metabarcoding is a targeted approach in which only specific markers within taxonomic groups of interest are sequenced, it is more cost‐effective than metagenomics. There is therefore a trade‐off between metagenomics and metabarcoding in the amount of data obtained both across and within taxa and the cost of sequencing, and thereby in how many samples can be screened. This trade‐off is important in animal diet studies that require many samples to be processed, for instance, in the case of vampire bats where many samples need to be screened to assess disease transmission risks at large geographic scale. However, in the present study, the use of a taxonomically broad metabarcoding marker allowed us to detect non‐target diet taxa such as intestinal parasites and arthropods, while allowing us to discern diet even to the species level and determine vampire bat population structure. This shows that while metabarcoding arguably does not generate as comprehensive data as metagenomics, with thoughtful primer selection it is possible to use its capacity to process many samples while obtaining more data than presence–absence of expected diet taxa. In the following, we discuss our metabarcoding methodology to enable future metabarcoding diet studies to couple information on diet and population structure, we discuss the use of this approach to screen common vampire bat bloodmeal and faecal samples, and we discuss the specific findings regarding the common vampire bat in Peru.

### 4.1 Metabarcoding diet analyses of the common vampire bat

We detected a high proportion of vampire bat DNA compared with prey DNA, and our results indicate that the prey DNA was fragmented, as the relative abundance of common vampire bat sequences was generally higher and prey detection lower for the longer marker (COI) (Figure 2). These observations are not uncommon in metabarcoding diet studies. Predator DNA is generally in excess, even to the point that it swamps the prey DNA (e.g., Deagle et al., 2006; Shehzad et al., 2012). One approach to reduce PCR amplification of predator DNA is to use predator blocking primers (Deagle et al., 2009; Vestheim & Jarman, 2008), although coblocking of prey has been reported (Piñol, Mir, Gomez‐Polo, & Agustí, 2015). We could not design common vampire bat‐specific blocking primers due to high intraspecific variation in the common vampire bat and the lack of reference sequences for potential prey species. Instead, we optimized amplification of prey DNA in the metabarcoding PCRs. Both low amounts of template and PCR inhibitors (such as co‐extracted heme compounds, Akane, Matsubara, Nakamura, Takahashi, & Kimura, 1994) can limit PCR amplification, and thereby sequencing, of taxa that are in low proportions and/or are not well amplified by the primers (Chandler, Fredrickson, & Brockman, 1997; Murray et al., 2015; Polz & Cavanaugh, 1998). Therefore, we carried out an initial qPCR screening to ensure that we added the maximum template amount to the metabarcoding PCRs without causing PCR inhibition (Murray et al., 2015; Shapiro & Hofreiter, 2012). Additionally, we used the qPCR to determine the minimum number of PCR cycles to be run in the metabarcoding PCR to minimize PCR stochasticity and bias against poorly amplified taxa (Piñol et al., 2015; Polz & Cavanaugh, 1998). Furthermore, we used relatively short mitochondrial markers to optimize detection of fragmented prey DNA (Deagle et al., 2006), and we used two different markers in order for their prey detections to supplement each other. For each marker, three PCR replicates were made per sample to reduce effects of PCR stochasticity and optimize prey detection and error removal during bioinformatic processing (Alberdi et al., 2018; Polz & Cavanaugh, 1998). These measures enabled us to overcome challenges in using metabarcoding to assess the diet of a blood‐feeding predator that is in the same taxonomic group as its prey, without the use of blocking primers and to detect vertebrate prey in 93.2% of samples (Table 1), which far exceeds previous DNA‐based vampire bat diet studies that detected prey in 25%–50% of samples analysed (Bobrowiec et al., 2015; Carter et al., 2006; Ito et al., 2016).

Consistent with earlier studies, we identified both domestic and wild animals in the diet of the common vampire bats, but generally found domestic animals to be the main prey (e.g., Bobrowiec et al., 2015; Carter et al., 2006; Ito et al., 2016; Streicker & Allgeier, 2016; Voigt & Kelm, 2006), which is unsurprising given the high density and accessibility of domestic animals. In our study, mammal prey by far outweighed chicken detections (Figures 1b and 3). Furthermore, when comparing detected prey with numbers of available vertebrate prey, we found fewer chicken detections in the prey than what would be expected based on chicken availability, while cows and pigs were overrepresented in the diet compared with availability (Table S1d), although these analyses do not account for differences in biomass. These findings are in agreement with those of a study in Brazilian Amazonas that found that even though chicken was the most attacked prey, pigs were highly preferred in relation to prey availability, which suggested a preference for mammalian prey (Bobrowiec et al., 2015). However, when we compared preferences for only cow, pig and sheep, the bats did not have obvious preferences for pigs (Table S1e). While avian DNA was rare in common vampire bats and restricted to chickens in our study (Figure 3), three additionally analysed samples from hairy‐legged vampire bats only contained birds, including both domestic chickens and two wild bird taxa, the Spix's guan (*Penelope jacquacu*) and tinamous (*Tinamus* sp.) (Supporting Information S2). To our knowledge, birds other than chicken have not previously been identified in any vampire bat species’ bloodmeal or faeces.

Few wildlife species have previously been identified among the prey of vampire bats and all exclusively in the common vampire bat, namely sea lions (Catenazzi & Donnelly, 2008), lowland tapirs (*Tapirus terrestris*) and red brocket deer (*Mazama americana*) (Galetti, Pedrosa, Keuroghlian, & Sazima, 2016). We identified tapir (*Tapirus* sp.) in bloodmeals from four of 13 analysed bats in the MDD130 site in the Amazon (Figure 1). Based on the employed markers, we could not determine which tapir species the bats had fed on, but geographic distributions of tapir species indicate that it can have been lowland tapir (*Tapirus terrestris*). Identifying tapir in about a third of the bats at this site indicates that tapirs might be a reliable and accessible food source for the vampire bats here. Tapirs are commonly hunted by Amazonian communities where they occur and can be rapidly depleted due to their slow reproductive rates (Peres, 2001). If the bats rely on tapir, this creates a potential conflict between bats and humans for food; if tapir populations are depleted by human hunting, bats might shift to other wild mammals or humans, which would have consequences for disease transmission patterns and risks. As mentioned, we also detected wildlife in two of three additionally analysed bloodmeal samples from hairy‐legged vampire bats (Table S2), which further indicates the bats’ dependence on wildlife in this site. Although we did not detect human DNA, a previous study at this site recorded frequent bat bites on humans (Streicker & Allgeier, 2016). Currently, no other method exists that can identify domestic and wild prey to low taxonomic levels across large numbers of vampire bats. Thereby, metabarcoding enables future studies to, for example, assess how much vampire bats rely on co‐occurring wildlife, how bat diet, and consequently risk of disease transmission, is altered following, for example, hunting or environmental change and to determine vampire bat vertebrate prey taxa over large geographic scales and use it to inform projections of disease transmission.

Apart from vertebrate prey, metabarcoding allowed detection of arthropods in the faecal and bloodmeal samples. Although vampire bats are thought to subsist only on vertebrate blood (Bobrowiec et al., 2015; Carter et al., 2006; Ito et al., 2016), sparse documentation of arthropods in common vampire bat stomachs does exist (Arata, Vaughn, & Thomas, 1967; Greenhall, 1972). This information was obtained through invasive measures, while our metabarcoding study allowed detection of arthropods without sacrificing the bats. Our study indicates that consumption of arthropods by common vampire bats is a widespread and relatively common phenomenon. Furthermore, metabarcoding confirmed the arthropods to be ectoparasitic taxa when DNA reference databases were sufficient to identify DNA to low taxonomic resolution. Our high observed frequency and geographically widespread nature of this phenomenon are unsurprising given that common vampire bats have high levels of infection by ectoparasites and engage in self‐ and allo‐grooming (Patterson, Dick, & Dittmar, 2008; Carter & Leffer, 2015; reviewed in Greenhall et al., 1983). Detected arthropods are therefore likely ectoparasites ingested during grooming (Aguirre, Herrel, & Van Damme, 2003; Greenhall, 1972). However, it is also conceivable that ectoparasites might be ingested while the bats feed from vertebrate prey, which if confirmed, would support the hypothesis that blood feeding in vampire bats might have evolved from a previous specialization on ectoparasitic arthropods of larger animals (Arata et al., 1967; Fenton, 1992; Gillette, 1975).

Lastly, it is worth mentioning factors which should be considered when interpreting the metabarcoding diet results. First, more than one individual prey can only be detected if they are different species. Second, multiple vampire bats can consume blood from the same prey individual, even on the same night (Greenhall, Schmidt, & Lopez‐Forment, 1971), meaning that detection of, for example, four tapir feeding events does not necessarily indicate that four tapirs were present and fed upon. Finally, prey detected in an individual bat might not be prey that was directly preyed upon by the bat itself as common vampire bats regurgitate and share bloodmeals with roost mates that did not manage to feed (Wilkinson, 1984). Despite these limitations, our results show that metabarcoding is an efficient tool for high‐throughput molecular characterization of variation in the feeding behaviours of vampire bats, and that it can reveal both vertebrate prey, arthropods and gut parasites if a broad taxonomic marker is chosen.

### 4.2 Metabarcoding to determine common vampire bat population structure

Whereas our study relied on second‐generation sequencing, previous studies on common vampire bat population structure have relied on Sanger sequencing (e.g., Clare, Lim, Fenton, & Hebert, 2011; Streicker et al., 2016). In contrast to Sanger sequencing that produces one sequence per marker per sample, metabarcoding produces thousands of sequences, of which some carry errors such as those arising during PCR and sequencing (see e.g., Alberdi et al., 2018). We used filtering approaches and OTU clustering to optimize reliability of the resulting sequences (Alberdi et al., 2018). But in contrast to OTU clustering with the aim to approximate species equivalents, we clustered at a high similarity to balance detection of intraspecific variation with identifying only real intraspecific variation. This metabarcoding methodology can be extrapolated for simultaneous assessment of diet and predator population structure in metabarcoding diet studies of other predators in which predator DNA is co‐amplified with the prey and where the marker has intraspecific variation for the predator.

In our study, both employed markers had clear geographic structure for the common vampire bat (Figure 4). The mitochondrial markers from all bats from the coastal sites west of the Andes were distinct from bats at other sites. For the 16s haplotype, they differed with 6 bp from the other identified 16s haplotype, and for the COI, they differed with 23 and 25 bp from the two other COI haplotypes (Figure 4 and S1b). This indicates that the Andes act as a barrier for dispersal, at least for female vampire bats. Mitochondrial geographic structure has been reported in the common vampire bat in the cytochrome b (Ditchfield, 2000; Martins, Ditchfield, Meyer, & Morgante, 2007; Martins, Templeton, Pavan, Kohlbach, & Morgante, 2009; Streicker et al., 2016) and COI region (Clare, 2011; Hernández‐Dávila et al., 2012). For the COI, the reported intraspecific sequence divergence is compared with what we detected. Some have suggested that high mtDNA differentiation indicates the presence of cryptic common vampire bat species (Martins et al., 2007), but less marked geographical structure in nuclear markers suggests that populations are not entirely isolated (Clare, 2011; Martins et al., 2009; Streicker et al., 2016). The high site‐specificity in mtDNA, but not in nuclear markers, can be explained by the social organization of the bat, where female offspring stay in their natal group unless their mothers die or move, while males disperse (Wilkinson, 1985). Streicker et al. (2016) studied population structure of the common vampire bat in Peru using a cytochrome b marker and revealed more haplotypes than the two 16s and three COI haplotypes detected in the present study (Figure 4). This can be explained by the higher intraspecific variability of the cytochrome b region (Hajibabaei, Singer, Clare, & Hebert, 2007; Tobe, Kitchener, & Linacre, 2011). If a more fine‐scaled haplotype assessment is needed in future studies, a cytochrome b marker should therefore be used. It is important to note that the caveat of using mitochondrial markers is that it limits inference of population structure to females. In the case of the common vampire bat that has male‐biased dispersal and where males have been linked to disease spread, this limits the power of mitochondrial markers for prediction of spread of vampire bat‐transmitted pathogens (Streicker et al., 2016). For this purpose, nuclear metabarcoding markers should be used to complement the mitochondrial population structure (Streicker et al., 2016). However, for other research questions and purposes, the ability to use metabarcoding to simultaneously screen many samples for diet and be able to infer mitochondrial population structure “free of charge” can open up novel insights. To achieve this obviously requires choosing a study animal (and populations) with intraspecific mitochondrial diversity within a relatively short marker. Furthermore, it requires careful primer selection to choose metabarcoding markers with the desired level of intraspecific diversity for the predator, while optimizing amplification of diet taxa.

### 4.3 Scaling up vampire bat studies

In the present study, most bloodmeal and faecal samples were collected from individual vampire bats caught in the wild, which is intrusive to bats and sets a limit on how many samples can be collected. However, a few pooled faecal samples were collected noninvasively from underneath roosting bats, and as we showed, prey can be identified from such samples (Table 1). Future studies could exploit this noninvasive sample collection technique to scale up vampire bat diet and population structure assessments to larger numbers of samples. However, if this method is pursued, it is worth keeping in mind that pooled data will preclude conclusions about the number of individual bats represented without further analysis of bat identities using higher resolution genetic markers. Furthermore, pooled data do not allow quantification of the relative proportion of prey consumed by individual bats.

### 4.4 Perspectives

Exemplified by the common vampire bat we show that metabarcoding diet studies can simultaneously assess predator population structure and a wide range of prey taxa. Adaptation of this methodology will enable future animal ecology studies to, to some extent, offset the trade‐off between metagenomics and metabarcoding by harnessing the ability of metabarcoding to process many samples cost‐effectively while extending detections beyond presence–absence of expected diet taxa. When designed carefully, this approach can thereby yield an efficient, scalable and noninvasive assessment of population structure and diet, which can potentially yield novel insights in future animal diet studies.

For the common vampire bat, we showcase metabarcoding as a method for large‐scale screening of diet taxa, importantly with a higher success rate than previous DNA‐based methods, and for simultaneous assessment of common vampire bat population structure. This methodology can be used to obtain novel and noninvasive insights into common vampire bat ecology through coupling of dietary findings and population structure and to inform projections of which species are at risk of spillover infection by vampire bat‐transmitted pathogens, such as rabies. Thereby, this analytical tool can aid development of strategies for control of vampire bat‐transmitted rabies to humans, wildlife and livestock. For instance with regard to vaccination of susceptible prey species, which relies on knowledge of local vampire bat–prey interactions.

## AUTHOR CONTRIBUTIONS

The study was conceived by D.S., G.J., K.B., M.T.P.G.; sample collection was performed by D.S.; data generation was carried out by K.B., L.S.B.N., M.N.; data were analysed by K.B. and S.G.; the manuscript was written by K.B.; and all authors contributed with edits and comments.

## DATA ACCESSIBILITY

Sequencing data and information on tag and primer combinations in libraries are available from the Dryad Digital Repository, https://doi.org/10.5061/dryad.ct65669.
