# Supplementary material for: Using DNA metabarcoding for simultaneous inference of common vampire bat diet and population structure
Source: Mol Ecol Resour. 2018 May 16;18(5):1050–63. doi: 10.1111/1755-0998.12891 (PMC6120510; doi:10.1111/1755-0998.12891)
Supplement: Supplementary file 1 [file MEN-18-1050-s001.pdf]

MOLECULAR ECOLOGY RESOURCES

Supplemental Information for:

Using DNA metabarcoding for simultaneous inference of common vampire bat diet and population structure

Kristine Bohmann, Shyam Gopalakrishnan, Martin Nielsen, Luisa dos Santos Bay Nielsen, Gareth Jones, Daniel G. Streicker, M. Thomas P. Gilbert

Table of contents

**Supporting Information S1: Metabarcoding of common vampire bat blood meal and faecal samples**	**Page 2-7**
Table S1a: Overview of analysed common vampire bat blood meal and faecal samples	Page 2
Table S1b: Samples with common vampire bat and vertebrate prey taxa assignments from metabarcoding analyses using the 16s and COI primer sets.	Page 3
Table S1c: Number of prey taxa identified within the common vampire bat samples that had prey taxa assignments in metabarcoding analyses using 16s and COI primer sets	Page 3
Table S1d. Vertebrate prey availability and prey eaten at the six areas included in the study	Page 4
Table S1e. Mammalian prey availability and prey eaten at the six areas included in the study	Page 5
Figure S1a. Species accumulation curve for vertebrate prey detected in common vampire bat blood meal samples through metabarcoding of blood meal and faecal samples with 16s and/or COI primer sets	Page 6
Figure S1b**:** Alignments of 16s and COI common vampire bat haplotypes	Page 7
**Supporting Information S2: Metabarcoding of blood meals from hairy-legged vampire bats**	**Page 8-10**
Table S2. Overview of the taxa identified in three blood meal samples from hairy-legged vampire bat (*Diphylla ecaudata*)	10
**References**	**10**

MOLECULAR ECOLOGY RESOURCES

Supporting Information S1: Metabarcoding of common vampire bat blood meal and faecal samples

Table S1a. Overview of the common vampire bat blood meal and faecal samples analysed. Blood meal samples were collected from individual bats, while faecal samples were either collected from individual bats or collected non-invasively underneath bat roosts.

Ecoregion	Area	Province	District	Site	No. samples: Blood meals	No. samples: Individual faecal droppings	No. samples: ~5 pooled faecal droppings
**Amazon**	Amazonas	Rodriguez de Mendoza	Chirimoto	AMA1	3		
	Madre de Dios	Manu	Huepetuhe	MDD130	13		
	Madre de Dios	Manu	Mazuko	MDD134	9		
			No. samples		25		
**Andes**	Apurimac	Chincheros	Rio Blanco	API1	10		
	Apurimac	Abancay	Abancay	API13	2		
	Apurimac	Andahuaylas	Pacucha	API9	2		
	Cajamarca	Cutervo	Rodeopampa	CAJ2	15		
	Cajamarca	Cutervo	Ambulco	CAJ3	10		
	Cajamarca	Jaen	San Ingacio	CAJ4	2		
	Huanuco	Tingo Maria	Tingo Maria	HUA1		1	1
	Huanuco	Ambo	Cayna	HUA2		2	1
	Huanuco	Huanuco	Molinopampa	HUA3		2	1
			No. samples		41	5	3
**Coast**	Lima	Huaral	Chancay	LMA10	8		
	Lima	Canete	Mala	LMA4	15		
	Lima	Huara	Huacho	LMA6	21		
			No. samples		44		
			**Total no. samples**		**110**	**5**	**3**

MOLECULAR ECOLOGY RESOURCES

Table S1b. Samples with common vampire bat and vertebrate prey taxa assignments from metabarcoding analyses using the 16s (Taylor 1996) and COI (Geller *et al.* 2013; Leray *et al.* 2013) primer sets.

		**Blood meal**	**Individual faecal samples**	**Pooled faecal samples**	**All samples**
Total samples		110	5	3	118
No. samples with vampire bat id	16s	108 (99.2%)	5 (100%)	3 (100%)	116 (98.3%)
	COI	110 (100%)	5 (100%)	2 (66.7%)	117 (99.2%)
No. samples with vertebrate prey id	16s	100 (90.1%)	4 (80%)	3 (100%)	107 (90.7%)
	COI	91 (82.7%)	4 (80%)	2 (66.7%)	97 (82.2%)

Table S1c. Number of prey taxa identified within the common vampire bat samples that had vertebrate prey taxa assignments in metabarcoding analyses using 16s and COI primer sets.

		**Blood meal**	**Individual faecal samples**	**Pooled faecal samples**
Min. - max. (average) no. vertebrate prey taxa per sample where prey is detected	16s	1-2 (1.03)	1 (1)	2-3 (2.33)
	COI	1-2 (1.02)	1 (1)	3 (3)

MOLECULAR ECOLOGY RESOURCES

Table S1d. Vertebrate prey availability (expected counts, calculated using livestock densities) and prey eaten (observed counts, detected as prey through metabarcoding) in the six areas included in this study, and for all sites combined. All p-values show significant differences between observed and expected prey counts ($p<0.05$). However, for all individual areas and all areas combined, there are expected counts for species under 5, and the results must be interpreted with care.

Area		Cow	Chicken	Pig	Sheep	Pearson's χ^2 statistic	p-value
AMA	Observed	3	0	0	0	127.93	1.16×10^{-29}
	Expected	0.069	2.828	0.088	0.015		
API	Observed	11	0	1	0	31.05	2.52×10^{-8}
	Expected	2.861	4.736	1.336	3.068		
CAJ	Observed	18	0	8	0	43.46	4.33×10^{-11}
	Expected	7.700	13.578	2.337	2.385		
HUA	Observed	5	0	4	3	29.66	5.15×10^{-8}
	Expected	0.935	7.194	1.422	2.449		
LMA	Observed	24	0	8	3	1121.46	7.16×10^{-246}
	Expected	0.613	33.799	0.361	0.226		
MDD	Observed	13	2	1	2	15.80	7.03×10^{-5}
	Expected	5.338	6.625	1.545	4.492		
All areas	Observed	74	2	22	8	740.54	4.57×10^{-163}
	Expected	7.471	86.910	4.639	6.985		

MOLECULAR ECOLOGY RESOURCES

Table S1e. Mammalian prey availability (expected counts, calculated using livestock densities) and prey eaten (observed counts, detected as prey through metabarcoding) in the six areas included in this study, and for all sites combined. All p-values show significant differences between observed and expected prey counts ($p<0.05$). However, for some areas, there are expected counts less than 5, and for these the results must be interpreted with care.

Area		Cow	Pig	Sheep	Pearson's χ^2 statistic	p-value
AMA	Observed	3	0	0	4.49	0.03
	Expected	1.201	1.530	0.269		
API	Observed	11	1	0	14.06	1.77×10^{-4}
	Expected	4.725	2.207	5.068		
CAJ	Observed	18	8	0	7.19	7.35×10^{-3}
	Expected	16.117	4.892	4.991		
HUA	Observed	5	4	3	4.69	0.03
	Expected	2.335	3.551	6.114		
LMA	Observed	24	8	3	4.67	0.03
	Expected	17.876	10.521	6.603		
MDD	Observed	13	1	2	7.60	5.82×10^{-3}
	Expected	7.508	2.174	6.319		
All areas	Observed	74	22	8	51.42	7.47×10^{-13}
	Expected	40.690	25.268	38.042		

MOLECULAR ECOLOGY RESOURCES

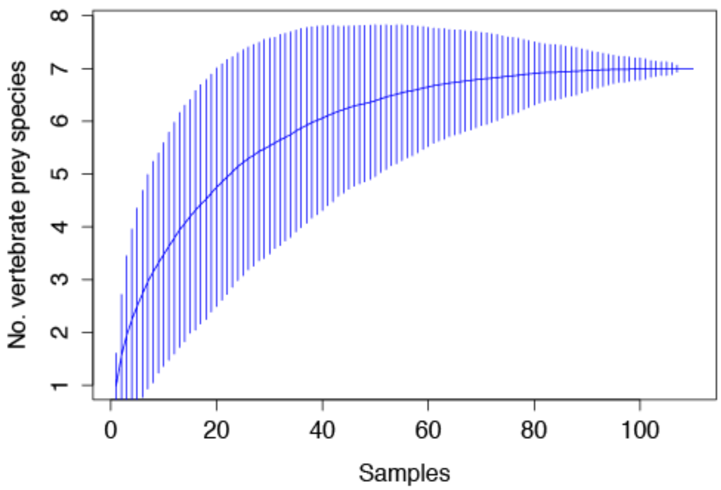

Figure. S1a. Species accumulation curve for vertebrate prey detected in common vampire bat through metabarcoding of blood meal and faecal samples with 16s and/or COI primer sets. Vertical bars indicate the bootstrap estimate of the standard error in the number of prey species detected.

MOLECULAR ECOLOGY
RESOURCES

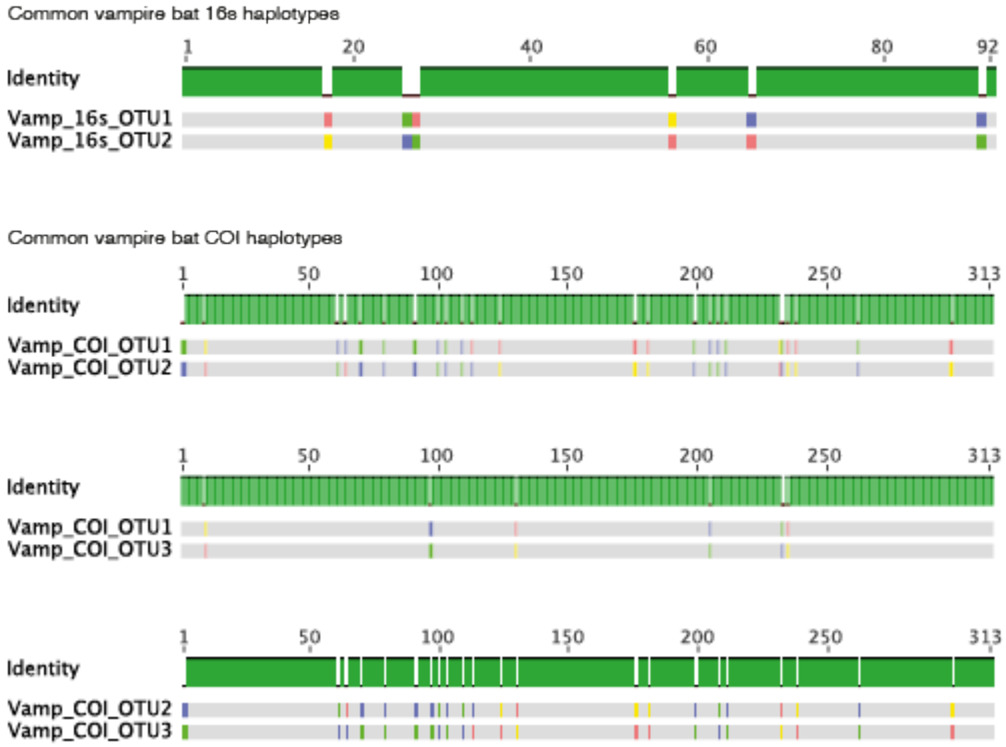

Figure S1b. Pairwise alignment of 16s and COI common vampire bat haplotype sequences. Image: Geneious version 6.1 created by Biomatters, http://www.geneious.com.

MOLECULAR ECOLOGY RESOURCES

Supporting Information S2: Metabarcoding of blood meals from hairy-legged vampire bats

Three hairy-legged vampire bat (*Diphylla ecaudata*) blood meal samples collected in the MDD134 site in the Amazon were metabarcoded alongside the common vampire bat (*Desmodus rotundus)* samples with the mammal 16s (Taylor 1996) and metazoan COI (Geller *et al.* 2013; Leray *et al.* 2013) primer sets. Blood meal samples were collected from captured and morphologically identified hairy-legged vampire bats. One hairy-legged vampire bat OTU was identified by each of the markers. The 16s OTU only had a 96% match to *Diphylla ecaudata* in ncbi genbank, while the COI hairy-legged vampire bat OTU had matches to *Diphylla ecaudata* spanning from 92.88-99.35% indicating mitochondrial intraspecific diversity in the hairy-legged vampire bat. As only bird prey was detected, only the metazoan COI primer set could be used to identify prey. With the COI primer set, four vertebrate prey OTUs were identified of which three were found in one sample and two samples contained the same OTU (Table S2). The BOLD database (boldsystems.org) was used to identify the OTUs. Criteria for taxonomic assignments were as follows: i) Prey species assignment: 100% match to only one species. ii) Prey genus assignment: 100% matches to more than one species within the same genus. As the remaining OTUs had poor reference database coverage, comparisons were made against all barcode records in BOLD. iii) Prey family assignment: identity from 96.12 to 88.03 within the same family. In the sample where Tinamous sp. (Tinamoformes) were identified (Table S2), an OTU with amount of sequences and low identity matches to the order Tinamiformes were also found. Therefore, this OTU was assumed to be an artefact. Nematode taxa assignment followed that of the common vampire bat.

Hairy-legged vampire bat has been reported to rely solely on birds as prey (Greenhall *et al.* 1984) (although potential predation on humans have been reported (Ito *et al.* 2016)). In agreement with this, we only detected birds in blood meal samples from three individual hairy-legged vampire bats. Two of the bats had preyed on chicken (*Gallus* sp.), while the third bat had preyed on two wild birds, spix's guan (*Penelope jacquacu*) and tinamous (*Tinamus sp.*) (Table S2). To our knowledge, no other study has identified other birds than chicken in any vampire bat species' blood meal or faeces. The spix's guan is a large, fairly common arboreal bird weighing up to ca 1.4 kg and known to occur in rainforest in the area where the samples were collected (IUCN 2016; Del Hoyo *et al.* 2017). Species in the tinamous genus are larger bird species weighing up to ca. 1.9 kg, they roost in trees, and the genus is known to occur in the area where the hairy-legged were caught (Khanna 2005; Del Hoyo *et al.* 2017). Although based on just one sample from one individual, this gives the first evidence that the hairy-legged vampire bats can prey on spix's guan and tinamous and that these wild birds might offer them a reliable and accessible food source in the MDD134 site.

MOLECULAR ECOLOGY RESOURCES

Table S2. Overview of the taxa identified through metabarcoding with the COI primer set (Geller *et al.* 2013; Leray *et al.* 2013) in three blood meal samples from hairy-legged vampire bat (*Diphylla ecaudata*) collected in the MDD134 site in the Amazon ecoregion. Vertebrate prey are listed as English name, order, family, genus and species.

Area, province, district	Site	No. blood meal samples	Vertebrate prey	Vertebrate prey	Additional detections
Madre de Dios, Manu, Mazuko	MDD134	1	**Spix's guan** Aves, Galliformes, Cracidae, *Penelope jacquacu*	**Tinamous** Aves, Tinamiformes, Tinamidae, *Tinamus* sp	
		1	**Chicken** Aves, Galliformes, Phasianidae, *Gallus* sp.		**Nematode** Nematoda
		1	**Chicken** Aves, Galliformes, Phasianidae, *Gallus* sp.		

References

Del Hoyo J, Elliott A, Sargatal J, Christie DA, de Juana E (2017) Handbook of the birds of the world alive. *Lynx Edicions*.

Geller J, Meyer C, Parker M, Hawk H (2013) Redesign of PCR primers for mitochondrial cytochrome c oxidase subunit I for marine invertebrates and application in all-taxa biotic surveys. *Molecular Ecology Resources*, **13**, 851–861.

Greenhall AM, Schmidt U, Joermann G (1984) *Diphylla ecaudata*. *Mammalian Species*, 1–3.

Ito F, Bernard E, Torres RA (2016) What is for dinner? First report of human blood in the diet of the hairy-legged vampire bat *Diphylla ecaudata*. *Acta Chiropterologica*, **18**, 509–515.

IUCN (2016) *Penelope jacquacu*: BirdLife International: The IUCN Red List of threatened species.

Khanna DR (2005) Biology of birds. *Discovery Publishing House*.

Leray M, Yang JY, Meyer CP *et al.* (2013) A new versatile primer set targeting a short fragment of the mitochondrial COI region for metabarcoding metazoan diversity: application for characterizing coral reef fish gut contents. *Frontiers in Zoology*, **10**, 34.

Taylor PG (1996) Reproducibility of ancient DNA sequences from extinct Pleistocene fauna. *Molecular Biology and Evolution*, **13**, 283–285.
